# Psychometric properties of the painDETECT questionnaire in rheumatoid arthritis, psoriatic arthritis and spondyloarthritis: Rasch analysis and test-retest reliability

**DOI:** 10.1186/s12955-017-0681-1

**Published:** 2017-05-22

**Authors:** Signe Rifbjerg-Madsen, Eva Ejlersen Wæhrens, Bente Danneskiold-Samsøe, Kirstine Amris

**Affiliations:** 1 The Parker Institute, Copenhagen University Hospital, Bispebjerg and Frederiksberg, 2000 Frederiksberg, Denmark; 2 The Research Initiative for Activity Studies and Occupational Therapy, The Research Unit of General Practice, Department of Public Health, University of Southern Denmark, Odense, Denmark; 3 Department of Rheumatology, Copenhagen University Hospital, Bispebjerg and Frederiksberg, Copenhagen, Denmark

**Keywords:** Inflammatory arthritis, Psychometrics, Rasch analysis, Test-retest reliability, PainDETECT questionnaire, Pain, Central sensitization

## Abstract

**Background:**

Pain is inherent in rheumatoid arthritis (RA), psoriatic arthritis (PsA) and spondyloarthritis (SpA) and traditionally considered to be of nociceptive origin. Emerging data suggest a potential role of augmented central pain mechanisms in subsets of patients, thus, valid instruments that can identify underlying pain mechanisms are needed. The painDETECT questionnaire (PDQ) was originally designed to differentiate between pain phenotypes. The objectives were to evaluate the psychometric properties of the PDQ in patients with inflammatory arthritis by applying Rasch analysis and to explore the reliability of pain classification by test-retest.

**Methods:**

For the Rasch analysis 900 questionnaires from patients with RA, PsA and SpA (300 per diagnosis) were extracted from ‘the DANBIO painDETECT study’. The analysis was directed at the seven items assessing somatosensory symptoms and included: 1) the performance of the six-category Likert scale; 2) whether a unidimensional construct was defined; 3) the reliability and precision of estimates. Another group of 30 patients diagnosed with RA, PsA or SpA participated in a test-retest study. Intraclass Correlation Coefficients (ICC) and classification consistency were calculated.

**Results:**

The Rasch analysis revealed: (1) Acceptable psychometric rating scale properties; the frequency distribution peaked in category 0 except for item 5, threshold calibration >10 observations per category, no disorder in the category measures for all items, scale category outfit Mnsq <2.0, small distances (<1.4 logits) between thresholds for category 1, 2 and 3 for all items. (2) The principal component analysis supported unidimensionality; the standardized residuals showed that 53.7% of total variance was explained by the measure and the magnitude of first contrast had an eigenvalue of 1.5, no misfitting items, clinical insignificant different item hierarchies across diagnoses (DIF < 0.5 logits). (3) A targeted item-person map, person and item separation indices of 1.88(reliability = 0.78), and 13.04 (reliability = 0.99). The test-retest revealed: ICC: RA 0.86(0.56–0.96), PsA 0.96(0.74–0.99), SpA 0.93(0.76–98), overall 0.94(0.84–0.98). Classification consistency was: RA 70%, PsA 80%, SpA 90%, overall 80%.

**Conclusion:**

The results support that the PDQ can be used as a classification instrument and assist identification of underlying pain-mechanisms in patients suffering from inflammatory arthritis.

## Background

Rheumatoid arthritis (RA), psoriatic arthritis (PsA) and spondyloarthritis (SpA) are considered systemic inflammatory rheumatic diseases that cause joint destruction, disability and pain. The traditional approach to pain management has focused on treatment of the underlying disease using anti-inflammatory, disease-modifying drugs [1, 2]. However, in some patients, pain does not improve despite seemingly good inflammatory control [3–7]. This suggests that although peripheral tissue inflammation significantly contributes to nociceptive pain generation in inflammatory arthritis [8], augmented central pain-processing may play a prominent role in persistent pain [6].Thus, there is a need for instruments that can assist in identifying patients with augmented central pain mechanisms and thereby help tailor an effective, individualised treatment.

To the best of our knowledge no instruments have been developed specifically to assist in mechanisms-based pain classification of patients with inflammatory joint disorders. The painDETECT questionnaire (PDQ) is a symptom-based assessment tool developed to assist identification of neuropathic pain [9]. It assigns a score to the patients, which classifies pain into three groups: neuropathic, unclear or non-neuropathic (nociceptive) pain. Neuropathic pain is characterized by allodynia, hyperalgesia, dysesthesia and sudden pain; somatosensory symptoms assessed by the PDQ [10]. Emerging evidence supports that there are striking pain phenotypic similarities between neuropathic pain and pain conditions characterised by augmented central pain processing; that is how patients express their symptoms of abnormal sensory perceptions and the quality of their pain [11]. Based on this overlap, the PDQ has been used as indicator of augmented central pain processing in patients with osteoarthritis and fibromyalgia [12–15] and recently, the PDQ has been introduced in studies of pain mechanisms in patients with RA [16, 17] and SpA [18]. Satisfactory psychometric properties of the PDQ have been demonstrated within osteoarthritis [19]. However, they remain to be evaluated within inflammatory arthritis and it is well-established that the psychometric properties of a questionnaire may vary depending on the population it is used in [20]. Because the PDQ is gaining ground as pain phenotyping instrument within these diagnoses, the psychometric properties, should be investigated as a prerequisite to implementing the PDQ in clinical research or daily practice, in order to secure valid and reliable pain classification of these particular patients. Rasch analysis allows detailed analyses of an instruments’ rating scale structure and measurement properties and as such it also gives important information for evaluation of the trichotomous classification system of the PDQ. Rasch analysis has traditionally been applied to questionnaires to evaluate a hierarchy of the items e.g. in the SF-36 [21], but it has also been shown to be useful in questionnaires where the items are equally descriptive of the condition as in the PDQ [22]. Furthermore, Rasch analysis includes a mathematical reliability measure describing how well an instrument differentiates between groups; patients with different pain phenotypes. Finally, large variation in the examined population e.g. with regard to disease severity and gender can ensure generalisability [23].

Whether an instrument has reliable classification ability, can be further investigated by test-retest, which will give an estimation of the stability [20]. Pain phenotypes are as such not expected to alter, thus the classification thereof should be consistent. Estimation of intraclass correlation coefficients based on exact scores can further support reliablity.

The overall purpose of this study was to evaluate the psychometric properties of the PDQ in patients with inflammatory joint disease (RA, PsA, SpA). Specifically, to conduct Rasch analysis, including reliability analysis of pain classification by means of person and item distinction in a sample of patients representing all diagnoses, both genders and every degree of activity of the disease. Further, to explore the agreement of scores and the stability of pain classification by test-retest.

## Methods

### Study design and setting

The Danish DANBIO-registry is a nationwide rheumatologic clinical quality registry, which covers >90% of adults treated with biologics due to rheumatic disease [24]. The registry also includes data on patients treated with synthetic disease-modifying antirheumatic drugs (DMARDs). Before routine clinical control of the arthritic condition, the patients regularly complete diagnosis specific patient reported outcomes on the DANBIO touch screens in the doctors waiting room. The rheumatologist registers corresponding objective clinical outcomes, hence common rheumatologic disease activity measures such as the disease activity score-28 joints (DAS28) [25, 26] and ankylosing spondylitis disease activity score (ASDAS) [27] are available. These scores are composite scores including subjective and objective parameters of level of inflammatory activity and global health and thus describe the current degree of activity of the specific disease.

Data for the Rasch analysis was acquired from ‘The DANBIO painDETECT study’ (in review), which was a cross-sectional survey describing the prevalence of pain phenotypes among patients with inflammatory arthritis in Denmark. After feasibility was demonstrated during a piloting period of a month at Frederiksberg Hospital, an electronic version of the PDQ was implemented nationwide on the DANBIO touch screens for 6 months (1 Dec 2013–1 June 2014). The test-retest study took place at the Department of Rheumatology, Frederiksberg Hospital, where patients with the diagnoses RA, PsA or SpA registered in DANBIO were randomly invited to complete the PDQ twice on the DANBIO touch screens in connection with a clinical control. Based on clinical opinion the retest should take place within ≥1 and <5 days after the first completion in order to reduce the influence of change in the underlying disease (ongoing peripheral inflammation) [20].

### Participants

To be eligible for inclusion in the Rasch analysis the PDQ had to be complete and originate from a patient who had a disease activity score registered on the same day as the questionnaire was completed. Given the very large sample, we were able to include questionnaires from patients with different degrees of disease activity. Seeking to ensure generalizability, it was decided that: 1) the extraction of questionnaires should be stratified in thirds according to three disease activity categories (low, moderate and high) defined by DAS28 or ASDAS, and 2) there should be an equal distribution of gender within the single diagnosis and disease activity category. Finally, patients with PsA may suffer from either peripheral and/or axial involvement [28], hence both DAS28 and ASDAS were accepted as disease activity scores. In cases where both scores were reported, the highest score defined the disease activity category.

These criteria restricted the sample, primarily because of the relatively low number of patients having a high disease activity score (53 women with SpA had a high ASDAS), which led to the final extraction procedure: for each diagnosis within each disease activity category, complete questionnaires from the first 50 female respondents and the first 50 male respondents were included.

In all, a sample of 900 questionnaires from 300 participants with either of the diagnoses (RA, PsA, SpA) and 450 of each gender was compiled. Still, this high number is considered more than sufficient to obtain stable item calibrations in defined subgroups (diagnosis/disease, activity category/gender) with 99% confidence, given good targeting [29].

In the test-retest study, the sample size calculation for the Intraclass correlation coefficient (ICC) was based on the assumption that the observed ICC value across all diagnoses would be ≥0.95 [30], also aiming at a confidence interval narrow enough for the lower 95% confidence limit to be above >0.80. With 10 participants, the 95% CI around an ICC of 0.95 would correspond to 0.83–0.99. Accordingly, the inclusion for the test-retest study was open until 10 patients with each of the diagnoses RA, SpA or PsA had completed the painDETECT twice with no missing data. Patient’s disease activity was scored on the test-day.

### The painDETECT questionnaire (PDQ)

The PDQ was originally developed and validated for pain classification purposes [9] and has been translated into several languages, including Danish (www.pfizerpatientreportedoutcomes.com). The PDQ comprises 12 items. The first three assess current pain, strongest pain during the past 4 weeks, and average pain during the past 4 weeks on a 0–10 point numerical rating scale from “none” to “worst imaginable”. The fourth item includes a chart representation of four pain course patterns depicting persistence, fluctuation and attacks; the patients have to choose the one resembling their pain pattern the most. The fifth item display a mannequin on which the patients mark their area of pain and pain radiation also responding to a yes/no question about the presence of radiating pain. The remaining seven questions regarding the presence and severity of somatosensory signs and symptoms are rated on a six-category Likert scale (from never (0) to very strongly (5)): (1) *burning sensation in the painful areas*, 2) *tingling or prickling in areas of pain*, 3) *light touching is painful,* 4) *sudden pain attacks in areas of pain*, 5) *cold or heat is occasionally painful,* 6) *numbness in the painful areas,* 7) *slight pressure triggers pain*) [9, 14]. For diagnostic purposes, a validated algorithm is used to calculate a total score ranging from −1 to 38. Pain intensity ratings are not included in the total score. The selection of one of four pain course patterns contributes to the total score with a value ranging from −1 to 1; the absence/presence of radiating pain with a value of 0 or 2; and the presence and severity of evaluated somatosensory signs and symptoms with a value ranging from 0 to 35. The resulting total score classifies pain into three groups: a score > 18 indicates that the presence of a predominant neuropathic pain component is likely, a score of 13 to 18 is considered uncertain, and a score < 13 indicates that a neuropathic pain component is not likely present [9]. Used for classification purposes, the PDQ has a sensitivity and specificity of 84% (electronic version) in a mixed chronic pain population using clinician-assessed diagnosis of pain mechanism as a criterion based validity index [9].

### Statistics

#### Baseline characteristics

Group differences were calculated using the non-parametric Kruskal-Wallis test for ordinal and interval variables and Chi-square test (*n* < 5; Fisher’s exact test) for categorical variables. SAS software (version 9.3; SAS Institute Inc., Cary; North Carolina, USA) was used for the statistical analyses.

#### Rasch analysis

Seven of the nine PDQ questions which contribute to the scoring algorithm were included in the Rasch analysis. This was because: 1) the majority of points achievable on the scoring algorithm, 35 out of 38, originate from these questions, and 2) the scoring of the remaining two elements of the PDQ does not have a character applicable to Rasch analysis [19]. The item that assesses pain course consists of four different patterns. A score of −1 is assigned to one of the patterns, 0 to another and finally 1 to the remaining two. The item of presence of pain radiation is dichotomous and has a score of either 0 or 2. Thus, neither of the scorings of these two items can logically be converted into an ordinal scale. This was not taken into consideration in the original development of the questionnaire as Rasch analysis was not performed [9]. Furthermore, it has been demonstrated by Moreton et al. [19] that these items perform less satisfactorily in Rasch analysis. The Rasch analysis was carried out applying the Rasch computer program WINSTEPS 3.90.0 [31]. Statistics evaluating aspects of validity and reliability were generated, including fit of the data to the Rasch model assertions [23, 32]. The measures of severity of neuropathic pain symptoms and the item difficulty measures are expressed in logits (log-odds probability units) [33]. Rasch analysis procedures have been described in detail elsewhere [23, 32, 34], hence our description of the Rasch method used is brief.

The analyses were undertaken to assess: first, the performance of the six-category Likert scale (*rating scale properties*); second, whether the seven items defined a unidimensional construct ‘neuropathic pain’ (*unidimensionality*); third, the reliability and precision of pain classification by the seven items (*precision and reproducibility*).

It was decided that the partial credit model (PCM) [35] would be used should the data not fulfil the criteria for the rating scale model (RSM). Both models are used with polytomous data (i.e. data derived from response scales with more than two categories). The PCM assumes that the distance is not the same between different response categories.

Based on Linacre’s guidelines [36, 37], the performance of the Likert scale was addressed with a rating scale analysis initially per diagnosis, followed by an analysis including all diagnoses. Five properties were examined; the frequency distributions, threshold calibration (<10 observations per category), monotonic increase in category measures, scale category outfit (Mnsq), and order of thresholds.

Analysis of dependency was based on correlation statistics that was interpreted as follows: correlation > 0.7 high local dependency, <0.4 low local dependency [34].

To address whether the seven somatosensory items of the PDQ defined a unidimensional construct (i.e. neuropathic pain), a principal component analysis (PCA) of the standardised residuals was performed and the item and person goodness-of-fit statistics were examined [38, 39]. When analysing goodness of fit, underfit and overfit to the Rasch model were evaluated to identify poorly fitting items that needed removal [23]. Also infit and outfit statistics were taken into consideration [23]. Critical values for mean squares were calculated based on the sample sizes for the separate diagnosis (infit MnSq = 1.12, outfit MnSq = 1.35, ZSTD < 2) and overall (infit MnSq =1.06, outfit MnSq S = 1.2, ZSTD < 2) respectively [40]. Subsequently, an evaluation of differential item functioning (DIF) was performed investigating diagnosis, gender and disease activity separately. DIF occurs when the item difficulty estimates vary between groups and items exhibiting DIF therefore, may represent a threat to unidimensionality. Additionally, the hierarchical order of item difficulties across diagnoses was explored.

The precision and reproducibility of the item difficulty estimates and the neuropathic pain severity measures were evaluated by the overall separation and reliability indices. To obtain a desired reliability coefficient of 0.80 for replicability of person and item ordering [23], the separation indices must be at least 2.0, and the reliability index should be as close as possible to 1.0 (range 0.0–1.0) [41]. Further, the item-person map showing the threshold distribution of items and persons respectively was assessed. Match of the range of the two distributions was considered good targeting.

#### Test-retest reliability

The PDQ was originally designed as a classification tool, and not intended for outcome measurement, thus, the primary statistical analysis for test–retest reliability was based on Intraclass correlation coefficient (ICC) statistics (absolute agreement) [42]. IBM SPSS Statistics 19 software was used for these analyses. A priori, the interpretation of the results was defined as follows: values greater than 0.7 represent acceptable agreement, while values greater than 0.8 represent strong agreement and greater than 0.9 very strong agreement [42]. Finally, the classification consistency (i.e. proportion of no change in pain phenotype) was calculated, as the PDQ originally was designed as a classification tool.

## Results

### Study sample

In the original ‘DANBIO painDETECT study’ in all 15,978 patients were invited to participate in the survey. They were registered as having any form of RA, PsA, SpA or unspecific inflammatory arthritis (UA) (osteoarthritis not included). In all, 7054 (44.2%) patients completed the PDQ (RA; *N* = 3826. PsA; *N* = 1180. SpA; *N* = 1093. UA; *N* = 955), while 864 (5.4%) partially completed the PDQ, 6133 (38.4%) declined to participate, and 1927 (12.0%) registered themselves as ‘pain-free’ (excluded).

Of the 7054 complete questionnaires, 4853 were eligible for the Rasch analysis (RA; *N* = 3199. PsA; *N* = 921. SpA; *N* = 723). Due to the strict inclusion procedure the final sample was 900.

### Baseline characteristics

Baseline characteristics of the Rasch sample are shown in Table 1. Group differences were found across disease activity categories within all diagnoses. Four differences were found across diagnoses for the baseline test-retest sample including age, disease duration, treatment with DMARD and swollen joint count (Table 2).

**Table 1:** Baseline characteristics of the Rasch population stratified by disease activity level

	*low*	*n*	*moderate*	*n*	*high*	*n*	*p-value*
RA							
Age (yrs)	60(53.5–65)	100	61(53–68.5)	100	59(50–68.5)	100	0.84
Duration (yrs)	8(3–17)	93	5.5(2–14)	92	5(1–14)	92	0.03^a^
PDQ score	8(5–14)	100	12(7–18)	100	18(12–25)	100	<0.01
DMARD mono n (%)	65(74)	88	62(75)	83	54(70)	77	0.79
> 1 DMARD n (%)	23(26)	88	21(25)	83	23(30)	77	0.79
Biologics n (%)	38(38)	100	33(33)	100	31(31)	100	0.56
DAS28-crp	2.2(1.8–2.7)	100	3.9 (3.6–4.5)	100	5.7(5.4–6.25)	100	<0.01
TJC 28	0(0–1)	100	4(3–7.5)	100	13(9–18)	100	<0.01
SJC 28	0(0–0)	100	1(0–3)	100	5.5(2–9)	100	<0.01
VAS pain mm	25.5(13–41.5)	100	50(32.5–65)	100	75(57.5–86.5)	100	<0.01
VAS fatigue mm	29(18.5–56)	100	54(36.5–73.5)	100	77.5(57–92.5)	100	<0.01
VAS global mm	25.5(13.5–52.5)	100	58.5(41.5–74)	100	82.5(70.5–93.5)	100	<0.01
HAQ	0.5(0.125–1)	99	1(0.625–1.375)	99	1.75(1–2.25)	100	<0.01
Crp mg/l	3(1.5–5.5)	100	7(3–14)	100	23.5(11.5–39.5)	100	<0.01
IgM-RF n (%)	69(80)	86	64(79)	81	58(78)	74	0.96
Anti-CCP n (%)	31(54)	57	30(51)	59	19(42)	45	0.46
PsA							
Age (yrs)	50.5(42–58)	100	52(41.5–60)	100	52(44–60)	100	0.56
Duration (yrs)	6(3–9)	91	4.5(1–9.5)	92	6(3–9)	87	0.30
PDQ score	9(5–12)	100	14(9–18)	100	19(14.5–24)	100	<0.01
DMARD mono n (%)	69 (95)	73	53(83)	64	54 (81)	67	0.04
> 1 DMARD n (%)	4(5)	73	11(17)	64	13(19)	67	0.04
Biologics n (%)	55(55)	100	44(44)	100	41(41)	100	0.11
DAS28-crp	1.8(1.6–2.35)	92	2.8(2.4–3.55)	92	4.4(3.8–5.1)	90	<0.01
TJC 28	0(0–1)	94	1(0–3.5)	92	5(2–12)	91	<0.01
SJC 28	0(0–0)	93	0(0–0)	92	0(0–2)	90	<0.01^b^
BASDAI (0–100)	21.5(13–28)	98	52(41–64)	98	77(71–85)	99	<0.01
BASFI (0–100)	15.5(7–27)	98	45.5(27–57)	98	74(62–86)	99	<0.01
ASDAS	1.5(1.1–1.8)	97	2.8(2.5–3.1)	97	4(3.7–4.5)	98	<0.01
VAS pain mm	20.5(12–30)	100	50(39.5–63)	100	77 (68–88.5)	100	<0.01
VAS fatigue mm	27(15–38)	100	65(48–77)	100	85(75–93)	100	<0.01
VAS global mm	22(14–30)	100	59.5(46–74)	100	85.5(77–94)	100	<0.01
HAQ	0.375(0–0.625)	96	0.75(0.51.25)	95	1.5(1–1.875)	96	<0.01
Crp mg/l	2(1–4**)**	100	3(1.5–6)	100	6(3–14)	100	<0.01
HLA-B27 n (%)	7(54)	13	10(53)	19	7(58)	12	0.95
SpA							
Age (yrs)	45.5(36–53)	100	43.5 (35–52.5)	100	47(37–54)	100	0.52
Duration (yrs)	6(3–13)	94	4.5(2–9)	90	5(2–12)	89	0.14
PDQ score	8(5–13.5)	100	15(9–19.5)	100	17.5(10.5–22.5)	100	<0.01
DMARD mono n (%)	24(100)	24	21(84)	25	26(96)	27	0.06
> 1 DMARD n (%)	0 (0)	24	4(16)	25	1(4)	27	0.06
Biologics n (%)	78(78)	100	56(56)	100	54(54)	100	<0.01^c^
BASDAI (0–100)	22.5(14–34)	100	49(39–61)	100	72.5(65–83)	100	<0.01
BASFI (0–100)	16(10.5–31.5)	100	40.5(27–56)	100	67.5(49–83.5)	100	<0.01
ASDAS	1.7(1.3–1.90)	100	2.9(2.5–3.1)	100	4.1(3.8–4.5)	100	<0.01
VAS pain mm	21.5(12–35)	100	53.5(38–72)	100	75.5(65.5–86)	100	<0.01
VAS fatigue mm	31.5(17.5–48)	100	65(45.5–81)	100	83(71.5–92)	100	<0.01
VAS global mm	24.5(15.5–36)	100	61(46–78)	100	84(74–92)	100	<0.01
Crp mg/l	2(1–4)	100	3(2–7)	100	10(5–21)	100	<0.01
HLA-B27 n (%)	28(82)	34	20(63)	32	20(67)	30	0.17

Values are the median (interquartile range) or (n (%)). *P*-values are calculated by Kruskal-Wallis Test, Fishers exact test or Chi square. Except where indicated otherwise there are difference/no difference across all groups according to *p* value. ^a^no group difference between moderate – high disease activity *p* = 0.54, ^b^no group difference between low-moderate disease activity *p* = 0.31, ^c^no group difference between moderate-high disease activity *p* = 0.78.*DMARD* Disease Modifying Anti-Rheumatic Drug, *DAS28-crp* Disease Activity Score 28 –crp, *TJC* Tender Joint Count, *SJC* Swollen Joint Count, *BASDAI* Bath Ankylosing Spondylitis Disease Activity Index, *BASFI* Bath Ankylosing Spondylitis Functional Index, *ASDAS* Ankylosing Spondylitis Disease Activity Score, *VAS* Visual Analogue Scale, *HAQ* Health Assessment Questionnaire, *HLA-B27* Human Leucocyte Antigen-B27, *IgM-RF* Immunoglobulin M Rheumatoid Factor, *Anti-CCP* Anti–citrullinated protein antibodies

**Table 2:** Baseline characteristics of the test-retest population

	RA	PsA	SpA	*p*-value
	(*n* = 10)	(*n* = 10)	(*n* = 10)	
Female sex n (%)	8 (80)	5 (50)	4 (40)	0.266
Age (years)	65 (62–68)	58.5 (49–67)	47 (32–52)	0.005^a^
Duration (years)	15.5 (8–24)	8.5 (5–14)	4 (2–6)	0.017^b^
DMARD n (%)	9 (90)	9 (90)	2(20)	0.003
Biologics n (%)	6 (60)	7 (70)	8(80)	0.879
DAS28-CRP	3.9 (3.7–4.2)	3.7 (3.1–4.4)	-	0.519
TJC 28	5.5 (4–10)	6 (2–9)	-	0.791
SJC 28	1 (1–4)	0 (0–0)	-	0.020
BASDAI (0–100)	-	67 (34–83)	28 (14–43)	0.364
BASFI (0–100)	-	60.5 (37–89)	18 (13–32)	0.212
ASDAS	-	3.05 (2.4–3.8)	2.1 (1.1–2.7)	0.104
VAS pain mm	43 (26–53)	61.5(25–73)	24.5 (19–41)	0.331
VAS fatigue mm	65 (40–79)	63.5 (32–82)	43 (26–67)	0.397
VAS global mm	50 (34–63)	72 (32–87)	29.5 (22–57)	0.219
HAQ	1.25 (0.875–2)	-	-	-
Crp mg/l	5.5 (1–10)	4 (1–8)	3.5 (1–9)	0.939
HLA-B27 n (%)	-	2(100)(*n* = 2)	3(75)(*n* = 4)	1
IgM-RF n (%)	5(50)	-	-	-
Anti-CCP n (%)	5(50)	-	-	-

Except where indicated otherwise values are the median (interquartile range). *P*- values are calculated by Kruskal-Wallis or Fishers exact test. ^a^significant difference between only RA-SpA and SpA-PsA. ^b^significant difference between only RA-SpA. *DMARD* Disease Modifying Anti-Rheumatic Drug, *DAS28-crp* Disease Activity Score 28 –crp, *TJC* Tender Joint Count, *SJC* Swollen Joint Count, *BASDAI* Bath Ankylosing Spondylitis Disease Activity Index, *ASDAS* Ankylosing Spondylitis Disease Activity Score, *VAS* Visual Analogue Scale, *HAQ* Health Assessment Questionnaire, *HLA-B27* Human Leucocyte Antigen-B27, *IgM-RF* Immunoglobulin M Rheumatoid Factor, *Anti-CCP* Anti–citrullinated protein antibodies

### Rating scale properties

The likelihood ratio test indicated lack of fit to an interval model (RSM, *p* < 0.001). Accordingly, the analysis was continued using the PCM [35]. Initially, the diagnostic groups were analysed separately, and subsequently they were combined in one overall analysis. Only minor differences were found between diagnoses; the analyses revealed a frequency distribution that peaked in category 0 of the Likert scale except for item 5 (*cold or heat is occasionally painful*), which peaked in category 4 for RA and PsA, and category 3 for SpA. In the overall analysis, the distribution for item 5 peaked in category 4. In the diagnosis specific analysis, the threshold calibration showed >10 observations per category except for category 5 in items 3 (*light touching is painful*) and 4 (*sudden pain attacks in areas of pain*) for RA, in items 2 (*tingling or prickling areas of pain*), 3 and 4 for PsA, and in items 2 and 4 for SpA. In the overall analysis >10 observations were found in all categories. Monotonic increase of the category measures was found for all of the items, both in the diagnosis specific and in the overall analysis. With the exception of item 4 in category 5 (few counts) for SpA, the scale category outfit revealed a Mnsq <2.0, indicating no introduction of noise to the measurement in any category. Threshold disordering was found for items 1 (*burning sensation in the painful areas*), 3, 4, 6 (*numbness in the painful areas*) and 7 (*slight pressure triggers pain*) for RA; items 6 and 7 for PsA; item 2, 3 and 6 for SpA and items 2 and 6 in the overall analysis. Small distances (less than 1.4 logits) between thresholds were found in all analyses across all items for categories 1, 2 and 3 (hardly noticed, slightly, moderately) suggesting lack of distinction between categories. Correlations did not indicate dependency for the individual diagnoses or overall sample; RA (0.09– -0.33), PsA (−0.03– -0.37), SpA (0.03– −0.35), overall (−0.01– -0.35).

### Unidimensionality

**Fig. 1:**
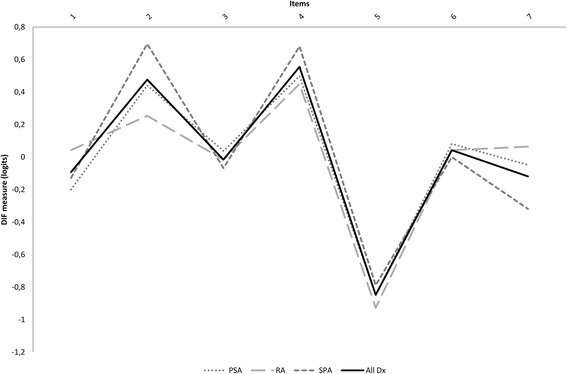
Differential item functioning (DIF) plot illustrating different item difficulty across diagnoses

The PCA of the standardised residuals revealed that 57.5% (RA), 52.6% (PsA), 53.1% (PsA) and 53.7% (overall) of the total variances were explained by the measures, respectively and that the magnitude of first contrast had an eigenvalue of 1.6 (RA, PsA, SpA) and 1.5 (overall) both supporting unidimensionality. Table 3 shows the fit statistics. Items 5 and 6 had mean square values above the infit criteria across diagnoses. DIF was observed within all areas tested; gender, diagnosis and disease activity, however the DIF contrasts were well below 0.5 logits [34]. Regarding gender, the DIF was related to items 3 and 4, where it was easier for men to obtain a high score, and items 5 and 6 where it was easier for women to obtain a high score. Furthermore, there was DIF across the three diagnostic groups on items 1, 2, 4 and 7 as illustrated in Fig. 1. This gives rise to different item hierarchies across diagnoses as shown in Table 4, where diagnosis specific item hierarchies based on item difficulty calibrations are presented. In the current analyses, items with a positive calibration were the most difficult (i.e. difficult to obtain a high score; least endorsed), whereas items distributed at the negative end of the scale were the easiest (i.e. easy to obtain a high score; most endorsed). Item number 5 had the same ranking in the hierarchy across all diagnoses being the easiest item, whereas items number 2 and 4 were the two most difficult items. Finally, there was DIF for disease activity for item 2 indicating that participants with high disease activity reported higher scores with regard to tingling or prickling in the painful areas.

**Table 3:** Item fit statistics

Mean square - Z standard deviation																
RA					PsA				SpA				All diagnoses			
Item	Infit		Outfit		Infit		Outfit		Infit		Outfit		Infit		Outfit	
1	0.82	−2.4	0.8	−2.3	1.04	0.6	1.06	0.7	0.91	−1.1	0.95	−0.5	0.92	−1.7	0.94	−1.2
2	0.93	−0.9	0.86	−1.5	0.72	−3.8	0.67	−3.4	0.84	−2.0	0.79	−1.8	0.84	−3.5	0.80	−3.5
3	1.04	0.5	1.01	0.2	0.99	−.01	0.99	−.01	1.02	0.3	1.01	0.2	1.01	0.2	1.00	0.1
4	1.01	0.2	0.97	−0.3	1.08	1.0	1.02	0.2	1.0	0.0	0.93	−0.6	1.03	0.6	0.97	−0.4
5	1.14	1.7	1.09	1.1	1.09	1.2	1.07	0.9	1.14	1.7	1.11	1.3	1.12	2.5	1.09	1.9
6	1.14	1.7	1.21	1.9	1.24	2.9	1.26	2.4	1.08	1.0	1.12	1.1	1.14	2.9	1.19	2.9
7	0.98	−0.2	0.99	0.0	0.91	−1.2	0.84	−1.8	1.04	0.6	1.05	0.6	0.99	−0.1	1.00	0.0

Critical values for mean squares were calculated based on the sample sizes for the separate diagnosis (infit MnSq = 1.12, outfit MnSq = 1.35, ZSTD < 2) and overall (infit MnSq =1.06, outfit MnSq S = 1.2, ZSTD < 2) respectively. Item1. Burning sensations; item 2. Tingling or prickling; item 3. Light touching painful; item 4. Sudden pain attacks; item 5. Cold or heat painful; item 6. Numbness; item 7. Slight pressure pain

**Table 4:** Item difficulty measures causing the different item hierarchies across diagnoses

Item difficulty			
RA	PsA	SpA	Across all diagnosis
5. Cold or heat painful (−0.93)	5. Cold or heat painful (−0.85)	5. Cold or heat painful (−0.79)	5. Cold or heat painful (−0.86)
3. Light touching painful (−0.02)	1.Burning sensations (−0.2)	7. Slight pressure pain (−0.32)	7. Slight pressure pain (−0.10)
1.Burning sensations (0.04)	7. Slight pressure pain (−0.05)	1.Burning sensations (−0.13)	1.Burning sensations (−0.10)
6. Numbness (0.04)	3. Light touching painful (0.04)	3. Light touching painful (−0.07)	3. Light touching painful (−0.02)
7. Slight pressure pain (0.06)	6. Numbness (0.08)	6. Numbness (0.00)	6. Numbness (0.04)
2.Tingling or prickling (0.25)	2. Tingling or prickling (0.44)	4. Sudden pain attacks (0.68)	2. Tingling or prickling (0.46)
4. Sudden pain attacks (0.45)	4. Sudden pain attacks (0.50)	2. Tingling or prickling (0.70)	4. Sudden pain attacks (0.54)

The easiest item is the most endorsed item (lowest difficulty measure); the most difficult item is the least endorsed item (highest difficulty measure)

### Precision and reproducibility

**Fig. 2:**
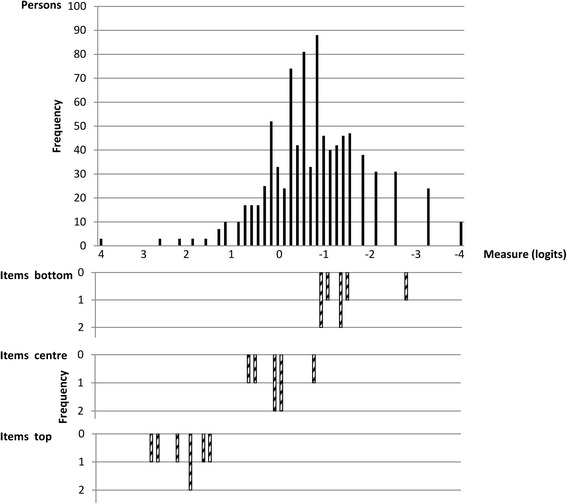
Item-person map illustrating the threshold distributions. The most difficult items and the person with most severe neuropathic pain are shown at the *left*. Each item is shown in three rows representing different rating scale measures. Items; *Center*: the mean item difficulty calibration. *Bottom*: measure level corresponding to a probability of 0.5 of being rated in the lowest category of the rating scale. *Top*: measure level coresponding to a probability of 0.5 of being rated in the highest category of the scale

The item-person distribution map, Fig. 2, illustrates that the items and participants were targeted and only 3 participants had maximum scores, thus no actual ceiling effect was observed. There was an indication of lack of easier items to capture the persons with less severe neuropathic pain symptoms. In the overall analysis which showed similar results as the diagnosis specific analyses, the person separation index was 1.88 (reliability = 0.78), and the item separation index was found to be 13.04 (reliability = 0.99).

### Test-retest reliability

**Fig. 3:**
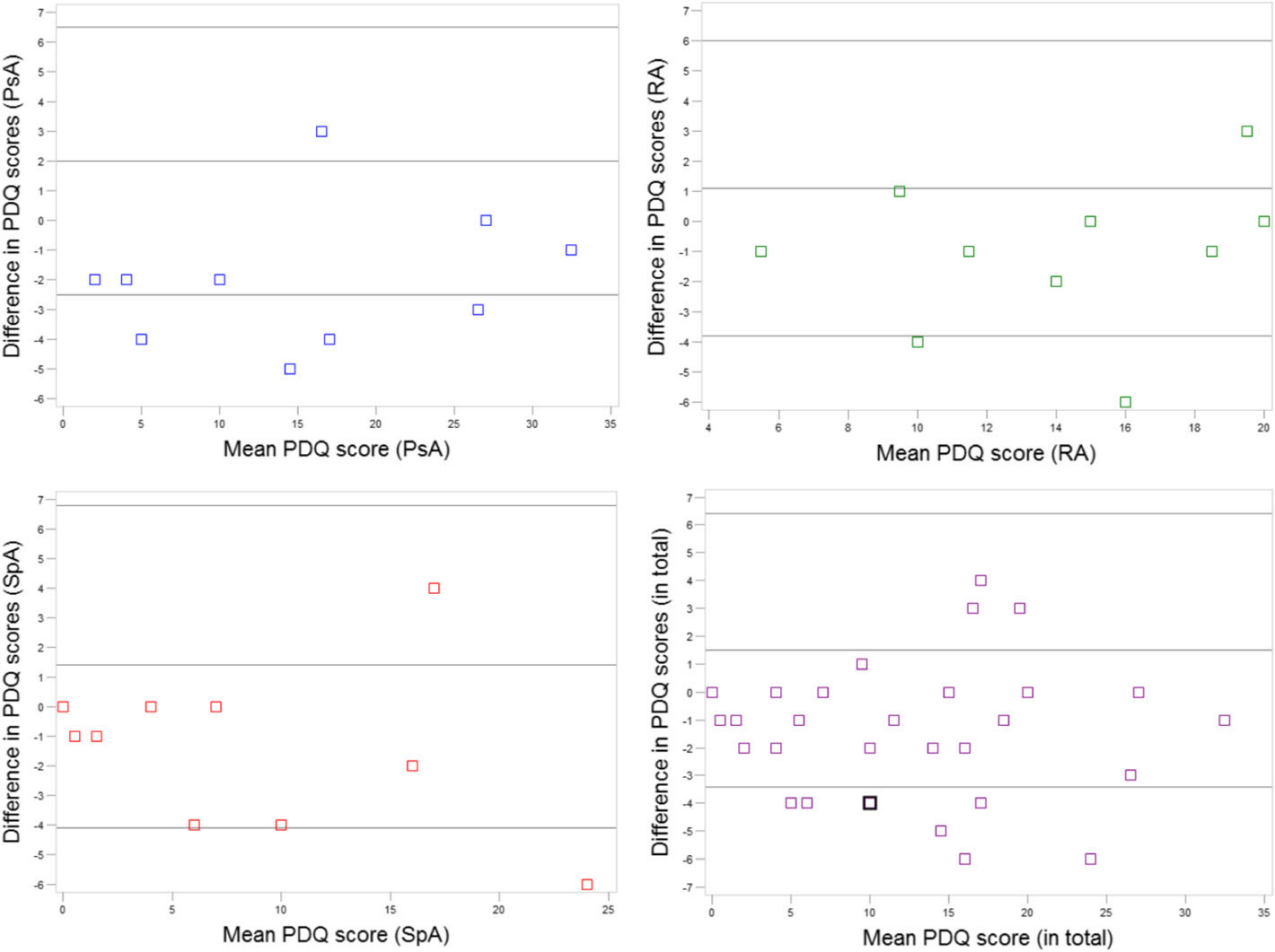
Bland Altman plots showing the agreement of PDQ scores between test and retest. ‘In total’ refers to the total of all scores across diagnoses. The square in bold in the diagram showing ‘in total’ represents two patients

Complete data were obtained from thirty participants, ten from each of the three diagnostic groups. The median (IQR) response time between test and retest was 2.5 (2–3) days. The median (IQR) PDQ ‘test’ scores were: RA 15(12–19), PsA 16 (7–27), SpA 7.5 (2–15), total 13.5 (7–19). The median PDQ ‘retest’ scores were RA 13 (10–18), PsA 13.5 (3–25), SpA 5.5 (1–15), total 11.5 (4–18). Intraclass correlation coefficients (ICC) were: RA 0.86 (0.56–0.96), PsA 0.96 (0.74–0.99), SpA 0.93(0.76–98) and total 0.94 (0.84–0.98). The Bland-Altman plots, Fig. 3, illustrate the differences between test and retest scores. The distribution of number of participants in the three PDQ classification categories according to diagnosis is reported in Table 5. Three, two and one participant with RA, PsA and SpA, respectively, changed classification group. This corresponded to a classification consistency of pain phenotype of 70% within RA, 80% within PsA, 90% within SpA, and 80% among participants in total. Those participants who changed group, changed between non-neuropathic and unclear (*n* = 1) or neuropathic and unclear (*n* = 5), with only few points between the first and second score.

**Table 5:** Distribution of participants in the PDQ categories according to diagnosis in the test-retest study

*n*						
	PDQ score < 13		PDQ score 13–18		PDQ score > 18	
	Test	Retestc	Test	Retest	Test	Retest
RA	4	4	3	4	3	2
PsA	4	5	2	2	4	3
SpA	7	7	2	1	1	2
In total	15	16	7	7	8	7

## Discussion

From the perspective of the Rasch model, the seven-item version of the PDQ assessing somatosensory symptoms of neuropathic pain demonstrated acceptable psychometric properties across all three diagnoses (RA, PsA and SpA), which was further supported by the test-retest analysis.

Current evidence suggests that both neuropathic pain and pain conditions characterised primarily by augmented central pain processing, e.g. fibromyalgia, may share similar neurobiological underpinnings [11]**.** Given that these mechanisms give rise to clinical symptoms, it may be expected that centrally mediated pain and neuropathic pain share clinical features, which are captured by neuropathic pain instruments based on somatosensory profiling such as the PDQ. Thus, the objective of this study was to investigate the psychometric properties of the PDQ when applied in pain classification in patients with RA, PsA and SpA. The study did not intend to evaluate construct validity, due to the lack of a clinically feasible and valid reference standard addressing this phenomenon.

The study showed overall acceptable rating scale properties of the PDQ supporting that the instrument may be used in pain classification; assigning patients to one of three categories based on a summed score and a validated algorithm. However, the rating scale analysis also demonstrated a potential for collapsing Likert-scale categories, as well as threshold disordering for items 2 and 6, in the overall analyses, which indicate that the summed PDQ score should be used with caution to grade the severity of neuropathic pain symptoms in inflammatory arthritis, in contrast to findings for osteoarthritis [19]. When focusing on unidimensionality, the test was satisfactory with all items representing the same construct ‘neuropathic pain’. DIF was found for different items for gender, diagnosis and disease activity. However, the magnitude of DIF contrasts was well below 0.5 logits, and hence had no clinical relevance [34]. This, together with the fact that the seven items of the PDQ assess the presence and severity of equally representative somatosensory symptoms of neuropathic pain, reduces the overall influence of the observed DIF on the total score, and it seems unlikely that it represented a threat to unidimensionality when the PDQ was used for classification of the construct ‘neuropathic pain’. Though unproven, there is a perception among some patients with inflammatory arthritis that warm conditions are related to symptom relief [43], which could explain the ranking of item 5 in the item hierarchies and the different use of the categories in the rating scale analysis.

The item–person map (Fig. 2) indicated that the PDQ had targeted items to the examined sample. No ceiling effect was found indicating that the items relevantly captured the neuropathic pain symptoms in the sample, the most severe included [23]. There was an indication of lack of easier items and subsequent lack of precision in the lower end of the scale, which may result from the design of the questionnaire only including items describing prototypical symptoms of neuropathic pain. With the exception of the item describing pain course pattern, no items describe prototypical symptoms of nociceptive pain, consequently the classification of nociceptive pain is based on the lack of neuropathic pain symptoms. This, we consider to be of less importance when the PDQ is being used to classify neuropathic pain. The indication of imprecision with regard to the item’s ability to distinguish between persons is probably explained by the same fact that the items are only describing symptoms of one phenomenon. High item separation was found, which may result from the very large sample size [23].

In the test-retest study, the ICCs reflected strong to very strong agreement depending on diagnosis while the Bland Altman plots showed some differences between test and re-test. In general the second score was lower than the initial score, which could be a result of a higher awareness of pain status, or actual reduction of symptoms, though this is not very likely having the short interval in mind. The PDQ is a classification instrument and accordingly we evaluated whether the difference in score that arose when the PDQ was administered repeatedly over days affected the classification group (pain phenotype). Consistent pain classification was obtained in 70% of participants with RA; 80% of participants with PsA; 90% of participants with SpA, and 80% of participants in total. This, in combination with the fact that no participants changed classification group between non-neuropathic and neuropathic pain indicated satisfactory consistency in pain classification.

### Strengths and limitations

The inclusion of a large and diverse sample of patients with inflammatory rheumatic joint disorders was a great strength of the Rasch analysis. The study sample was not representative for the general patient population encountered in daily clinical care, but was chosen to represent as large a variation as possible to enable determination of the validity of the PDQ across the spectra of diagnoses, gender and disease activity. A limitation of the Rasch analysis was that two of the items were not applicable in the Rasch model [19] due to their character, though this may have been handled to some extent by transformation into interval level data, this was not done as there was no intention to create a scale for measuring change and others have demonstrated difficulties in doing so [19]. However, the somatosensory profiling of pain is based on the seven items subjected to analyses [14, 44]. It might be questioned whether the cut off points of the scale validated in patients with various chronic pain conditions are applicable in patients with inflammatory arthritis. Ideally, future research should address the criterion validity and the cut off points of the PDQ scale by testing it against a clinical ‘gold standard’. Although this standard currently does not exist, it could be approached for instance by constructing clinical consensus on signs and symptomatology and the use and interpretation of quantitative sensory testing, test of descending pain pathways and neuro-imaging. The test-retest study was based on a priori power calculations of ICC and was therefore limited by a somewhat small sample size in relation to classification consistency. Furthermore, the study sample reflected the characteristics of the distinct diagnosis and correspondingly differences in baseline characteristics across the three diagnoses were found. Measurement of C-reactive protein is included in the disease activity score, and therefore evaluation of disease activity at retest was not feasible.

## Conclusion

In conclusion; from the perspective of the Rasch model, the seven-item version of the PDQ assessing somatosensory symptoms of neuropathic pain based on a six-category Likert scale, demonstrated sufficient psychometric properties when applied in a clinical sample of patients with RA, SpA and PsA. Consistency in pain classification was strong to very strong. It is therefore suggested that the PDQ may be used as an easily applied instrument assisting mechanism-based pain classification and identification of individuals with a significant central pain component, as strategies in addition to inflammatory disease suppression are likely to be required in the management of these patients.

Due to the observed relative problems with the rating scale, caution is urged in grading the severity of somatosensory symptoms in inflammatory arthritis.

## Abbreviations

ASDAS: Ankylosing spondylitis disease activity score
BASDAI: Bath ankylosing spondylitis disease activity index
BASFI: Bath ankylosing spondylitis function index
CRP: C reactive protein
DAS28-CRP: Disease activity score 28 joints C reactive protein
DIF: Differential item functioning
DMARD: Disease-modifying antirheumatic drug
FM: Fibromyalgia
GH: Global health
HAQ: Health assessment questionnaire
HLA-B27: Human leucocyte antibody, subtype B27
ICC: Intraclass correlation coefficient
IQR: Inter quartile range
Mnsq: Mean-square
PCA: Principal component analysis
PCM: Partial credit model
PDQ: PainDETECT questionnaire
PsA: Psoriatic arthritis
RA: Rheumatoid arthritis
RSM: Rating scale model
SJC: Swollen joint count
SpA: Spondyloarthritis
TJC: Tender joint count
UA: Unspecific arthritis
VAS: Visual analogue scale
